# Quantifying heterogeneous contact patterns in Japan: a social contact survey

**DOI:** 10.1186/s12976-019-0102-8

**Published:** 2019-03-20

**Authors:** Lankeshwara Munasinghe, Yusuke Asai, Hiroshi Nishiura

**Affiliations:** Graduate School of Medicine, Hokkaido University, Kita 15 Jo Nishi 7 Chome, Sapporo, Japan

**Keywords:** Epidemic, Epidemiological model, Mathematical model, Cumulative incidence, Influenza

## Abstract

**Background:**

Social contact surveys can greatly help in quantifying the heterogeneous patterns of infectious disease transmission. The present study aimed to conduct a contact survey in Japan, offering estimates of contact by age and location and validating a social contact matrix using a seroepidemiological dataset of influenza.

**Methods:**

An internet-based questionnaire survey was conducted, covering all 47 prefectures in Japan and including a total of 1476 households. The social contact matrix was quantified assuming reciprocity and using the maximum likelihood method. By imposing several parametric assumptions for the next-generation matrix, the empirical seroepidemiological data of influenza A (H1N1) 2009 was analysed and we estimated the basic reproduction number, *R*_0_.

**Results:**

In total, the reported number of contacts on weekdays was 10,682 whereas that on weekend days was 8867. Strong age-dependent assortativity was identified. Forty percent of weekday contacts took place at schools or workplaces, but that declined to 14% on weekends. Accounting for the age-dependent heterogeneity with the known social contact matrix, the minimum value of the Akaike information criterion was obtained and *R*_0_ was estimated at 1.45 (95% confidence interval: 1.42, 1.49).

**Conclusions:**

Survey datasets will be useful for parameterizing the heterogeneous transmission model of various directly transmitted infectious diseases in Japan. Age-dependent assortativity, especially among children, along with numerous contacts in school settings on weekdays implies the potential effectiveness of school closure.

**Electronic supplementary material:**

The online version of this article (10.1186/s12976-019-0102-8) contains supplementary material, which is available to authorized users.

## Background

The epidemiological dynamics of directly transmitted infectious diseases frequently exhibit highly heterogeneous patterns [1]. In particular, the transmission of acute infectious diseases tends to depend on the age of both primary cases and their contacts [2], indicating a critical need to account for age-dependent heterogeneity [3]. The basic reproduction number in such a heterogeneously mixing population is derived as the largest eigenvalue of the age-dependent next-generation matrix [1, 4], and its computation with *n* heterogeneously mixing age groups requires quantification of *n × n* elements of the matrix. However, it is frequently the case that the degrees of freedom during the statistical estimation are limited (e.g., limited by *n* different values of input data) [5].

Because use of the so-called WAIFW (who acquires infection from whom) matrix serves as an approximation of the actual heterogeneous contact pattern [2], this has recently attracted the attention of researchers, and conducting a social contact survey appears to greatly assist in reducing uncertainty with respect to heterogeneous contact patterns and quantifying a WAIFW matrix [6, 7]. In many instances, participants in social contact surveys, who are mostly recruited via convenience sampling, are asked to report the number of contacts they experience on a given day (e.g., a weekday or weekend day) with someone in the same or a different age group, the social setting of that contact, the type of contact (e.g., exchanging a few sentences or physical bodily contact), the duration of contact, and so on. A landmark study reporting the social contact patterns among eight different European countries was conducted as part of the so-called POLYMOD, a European Commission project [6]. Starting with the first study published in 2008, similar surveys have been increasingly conducted across the world, mostly using a written diary [2, 6, 8, 9] or remote sensing device [10–12]. Additional survey methods have also been reported, including the analysis of time-use data [13] and mobile phone network data [14]. Such empirical data appear to be an essential back-up for infectious disease modelling in the event of emerging infectious disease epidemics, such as the Ebola virus disease epidemic in West Africa in 2014–2016 [15, 16].

POLYMOD surveys have taken place in East and Southeast Asian countries and regions, including China, the Hong Kong Special Administrative Region, Japan, Taiwan, Thailand, and Vietnam [8, 9, 17–23]. Whereas the findings have mostly echoed those reported in Western countries (e.g., strong age assortativity, decreased contacts with age, and increased contacts with household size) [8], these studies can be expected to provide key information to parameterize infectious disease transmission in these countries. An existing study in Japan by Ibuka et al. [17] also successfully identified characteristics similar to those stated above; however, that study indicated a weak predictability of influenza using a contact matrix alone. Also, people aged 20–29 years were considered to have been under-represented in the published survey. The need remains for a similar data collection method but one that addresses the issue of validation using infectious disease data, possibly with better representation of the Japanese population.

The present study aimed to conduct a contact survey in Japan, offering estimates of contacts according to 5-year age groups, and validating the matrix using a seroepidemiological dataset of influenza.

## Methods

### Data collection

We conducted an internet-based questionnaire survey covering all 47 prefectures in Japan. Area sampling was conducted using the population size in Japan, determining the sample size by age and prefecture to be proportional to the actual age- and prefecture-specific distributions. The survey was conducted via a private company that has a collection of monitors to act as potential respondents across Japan, and published studies that rested on the internet survey of the same company are found elsewhere [24–26]. The present survey started with an advertisement that was notified among all registered monitors, and of these, a total of 1476 households have voluntarily decided to participate in the survey. As a consequence, a total of 2271 respondents were conveniently sampled to achieve a proportional sample of 0.015% in each 5-year age stratum and prefecture. The survey was conducted from 28 October to 1 December 2014, with manual validation of unclear responses continuing until 9 March 2015. Demographic variables other than age and prefecture of residence included sex, occupation and household size. Each respondent recorded all contacts during one weekday (Wednesday) and one weekend day (Sunday). A contact was defined as an exchange of three Japanese sentences or a physical touch on the skin. Each survey respondent was requested to keep a diary, recording each contactor’s age, sex, type of contact (i.e., conversation or physical), and the duration and location of contact. The questionnaire format was the same as that used elsewhere [6].

### Contact matrix

According to the ages of each respondent–contactor pair, reported contacts were grouped into 15 discrete age groups (0–4, 5–9, …, 65–69, and 70 years or older). These constituted the so-called social contact matrix {*m*_ij_} representing the rate of contact between an individual in age group *j* with individuals in age group *i* on a given day. To quantify {*m*_ij_}, we adopted an assumption of reciprocity, i.e., the mean number of contacts that an individual in age group *i* experiences with individuals in age group *j* is equal to the number that an individual in age group *j* experiences with individuals in age group *i*. Thus, we adjusted the asymmetry of the contact rate using the age-dependent population size. The mean contact rate {*m*_ij_} was estimated using the maximum likelihood method, as previously reported [7].

### Validation using influenza data

A key assumption when using the social contact matrix to model age-dependent heterogeneity of infectious disease transmission is that the age-dependent transmission matrix containing the contact frequency is informed by the social contact matrix. To capture age-dependent heterogeneity, we used the so-called next-generation matrix, *K* = {*k*_*ij*_}, comprising the average number of secondary infections in age group *i* that are caused by a single infectious individual in age group *j* in a fully susceptible population. The matrix describes the per-generation heterogeneity for the risk of infection by age; in the simplest terms, it may be parameterized using the social contact matrix as *k*_*ij*_ = *qm*_*ij*_, where *q* is a constant that can be interpreted as the disease-specific infectivity [27]. Alternatively, one can parameterize *K* as *k*_*ij*_ = *s*_*i*_*m*_*ij*_ if the susceptibility varies with age group *i*, and similarly, *k*_*ij*_ = *u*_*j*_*m*_*ij*_ if the infectiousness of age group *j* matters biologically.

Once the parametric assumption of the next-generation matrix was determined, we quantified the next-generation matrix by analysing the empirical seroepidemiological dataset of influenza A (H1N1) 2009 collected by the government of Japan [28] and estimating the basic reproduction number, *R*_0_, which is interpreted as the average number of secondary cases generated by a typical single primary case in a fully susceptible population and is calculated as the largest eigenvalue of the next-generation matrix [1, 3, 4]. Depending on the parametric assumption, we estimated the constant *q* or age-dependent susceptibility *s*_i_ to scale the next-generation matrix. To do so, we analysed the age-specific final size distribution (or serologically determined cumulative incidence) of influenza A (H1N1) 2009, using the difference in the age-specific seroprevalence between the 2009 and 2010 surveys. In 2009 and 2010, a total of 6626 and 6539 samples were collected, respectively, from all age groups. In each year, the survey took place from June to September; thus, the surveys in 2009 and 2010 contained the first wave of the pandemic within the inter-survey period. To determine seropositivity, we imposed a cut-off level of 1:20 as the default [29]. Additional file 1: Table S1 shows the dataset used to validate the importance of using the social contact matrix. Subtracting the positive fraction in 2009 from that in 2010, we obtained the empirical value of cumulative incidence *z*_i_, which satisfies the so-called age-dependent final size equation:

$$ {z}_i=1-\exp \left(-{\sum}_j{z}_j{k}_{ij}\right) $$

where *i* and *j* denote age groups and {*k*_*ij*_} denotes the next-generation matrix. Iteratively solving this equation, we optimized the likelihood function

$$ L\left(\theta; \boldsymbol{n},\boldsymbol{m}\right)=\prod \limits_i\left(\begin{array}{c}{n}_{10,i}\\ {}{m}_{10,i}\end{array}\right){\left({z}_{9,i}+{z}_i\right)}^{m_{10,i}}{\left(1-{z}_{9,i}-{z}_i\right)}^{n_{10,i}-{m}_{10,i}} $$

where *n*_10,i_ and *m*_10,i_ represent the observed total and positive serological samples in age group *i* in 2010, respectively, *z*_9,i_ is the positive fraction of age group *i* in 2009, and *θ* is the population parameter. We parameterized the next-generation matrix in various ways and tested the goodness of fit referring to the Akaike information criterion (AIC). As possible candidate matrices that do not rest on the social contact matrix, we tested the fully parameterized next-generation matrix including the homogeneous mixing assumption (*k*_*ij*_ = *R*), where *R* is a constant and the separable mixing assumption (*k*_*ij*_ = *a*_*i*_*a*_*j*_), comparing their fit against those using the social contact matrix. The 95% confidence intervals (CI) of parameters were computed using the profile likelihood.

### Ethical considerations

The purpose of the study was explained to participants, and they were ensured that the extent of the use of survey information would be limited to the present study. Informed consent was obtained via a website, and participants had the right to withdraw at any time during the study period. The Medical Ethics Committees of the Graduate School of Medicine, The University of Tokyo approved this study (approval ID: 10478). As for the seroepidemiological data, the present study used publicly available data [28]. The datasets had already been fully anonymized and did not include any identifiable information. Thus, ethical approval was not required for the analysis of seroepidemiological data.

### Data sharing policy

Seroepidemiological surveillance data can be accessed online via a linked URL [28]. A summary of the contact datasets presented in this study can be obtained from the corresponding author upon request.

## Results

**Fig. 1:**
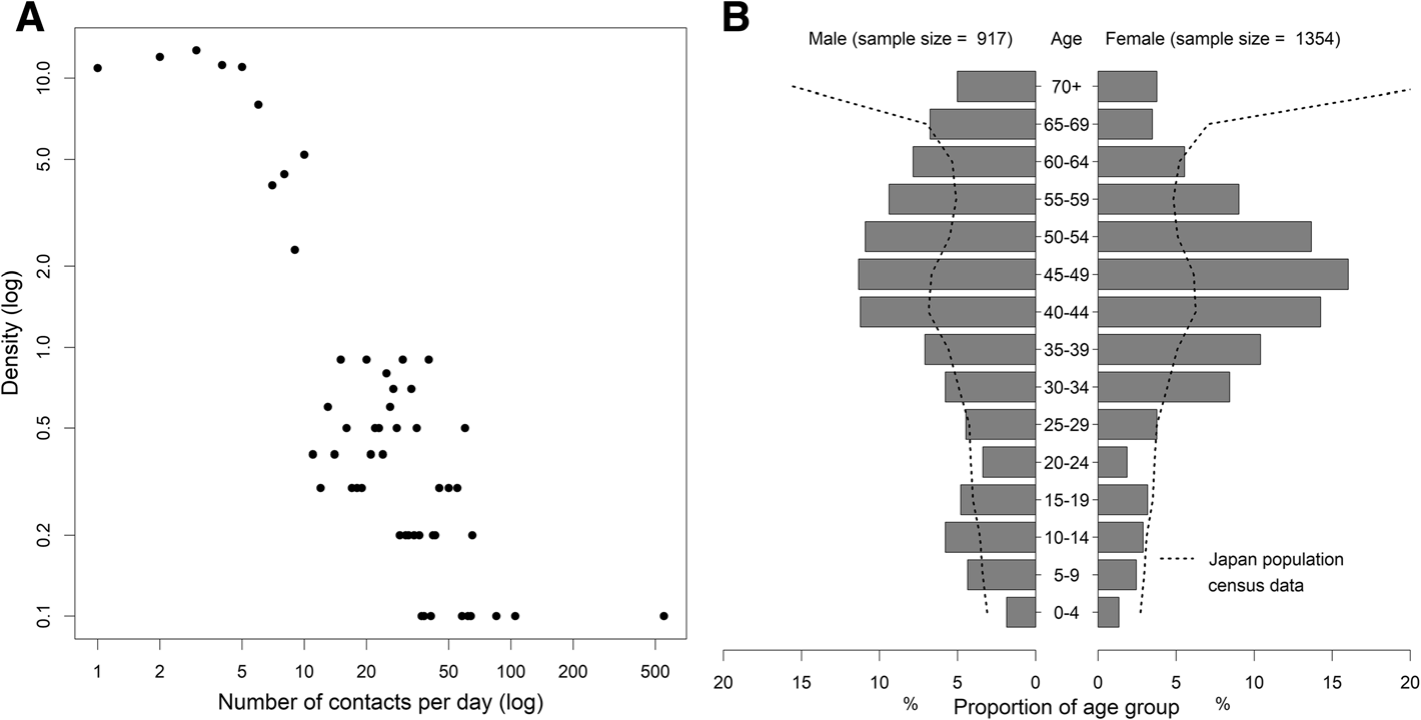
Distributions of contact frequency and age of sample population in Japan. **a** Log-log plot of contact frequency distribution. Logarithm of the proportion of the sample population was taken against the number of contacts (contact frequency) per day. **b** Age distribution of the study sample (bars) by age group and sex. Dashed lines represent the age distributions of the entire population of Japan as of 1 November 2016, overlaid with the sample population

In total, the reported number of contacts on weekdays was 10,682 and that on weekend days was 8867. Figure 1a shows the distribution of the daily rate of contacts per person, revealing a right-skewed distribution that could potentially be approximated to follow a power law, which can be confirmed by approximately a linear distribution by log-log plot. Due to area sampling with a proportional age sampling distribution, the samples of our survey qualitatively captured the essential part of the ages that were observed, whereas the ages among elderly respondents were relatively under-sampled (Fig. 1b). Due to the internet survey that starts with recruitment of registered monitors that are dominated by housewives, female slightly dominated respondents (59.6%). Common occupations included office workers (25.4%), housewives (23.9%), part-time worker (12.4%), retired persons (4.9%), primary school children (4.5%), and self-employed persons (4.2%). The household size ranged from 1 to 10 with the mean and standard deviation of 3.2 and 0.2 persons, respectively, which are consistent with the household size of the entire Japan.

Of contacts on weekdays and weekend days, 27.8 and 34.9% were classified as physical contacts, respectively. On weekdays, the highest average number of physical contacts per person (5.36) was seen in the age group 0–4 years, and the highest number of non-physical contacts (5.67) was seen in respondents aged 15–19 years. The lowest average number of physical contacts (0.69) was found among those aged 65–69 years and that of non-physical contacts (0.12) was among small children aged 0–4 years (Additional file 2: Table S2). These patterns were maintained on weekend days (Additional file 3: Table S3). The difference between weekdays and weekend days was highlighted by the location of contact, i.e., 40% of weekday contacts took place at school or the workplace, but this declined to 14% on the weekend (Additional file 4: Figure S1). On the contrary, household contacts accounted for 35 and 50% of contacts on weekday and weekend days, respectively.

**Fig. 2:**
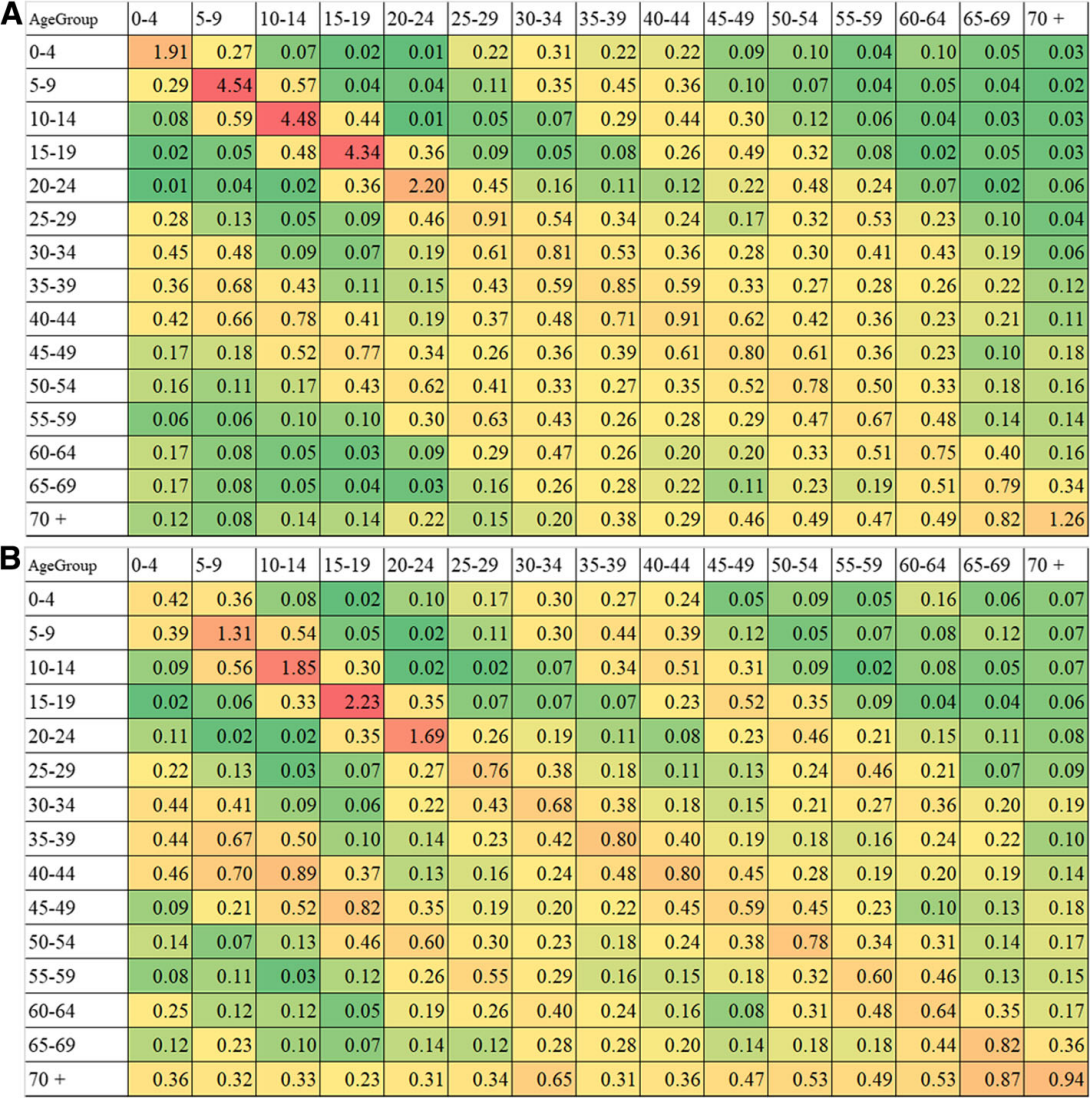
Contact matrix on weekdays and weekend days. **a** Average weekday and **b** weekend contact rate with discrete gradations. Age-dependent contact heterogeneity is approximately captured by these matrices. The number in each cell represents the contact rate per person

An age-dependent contact matrix by 5-year age groups is shown in Fig. 2. The assortativity coefficient, measured using Newman’s assortativity index or equivalently, Pearson’s moment correlation, was 0.27 and 0.18 for weekday and weekend contacts, respectively, clearly indicating that the mixing pattern was highly age assortative. From this difference and the data in Additional files 2 and 3: Tables S2 and S3, it can be seen that the assortative contacts stemmed from contacts taking place at schools or workplaces. In addition to the contacts within similar age groups, we identified the relatively high contact between children and those aged from 25 to 40 years, representing household contacts. The density of household contacts was elevated on weekend days.

**Fig. 3:**
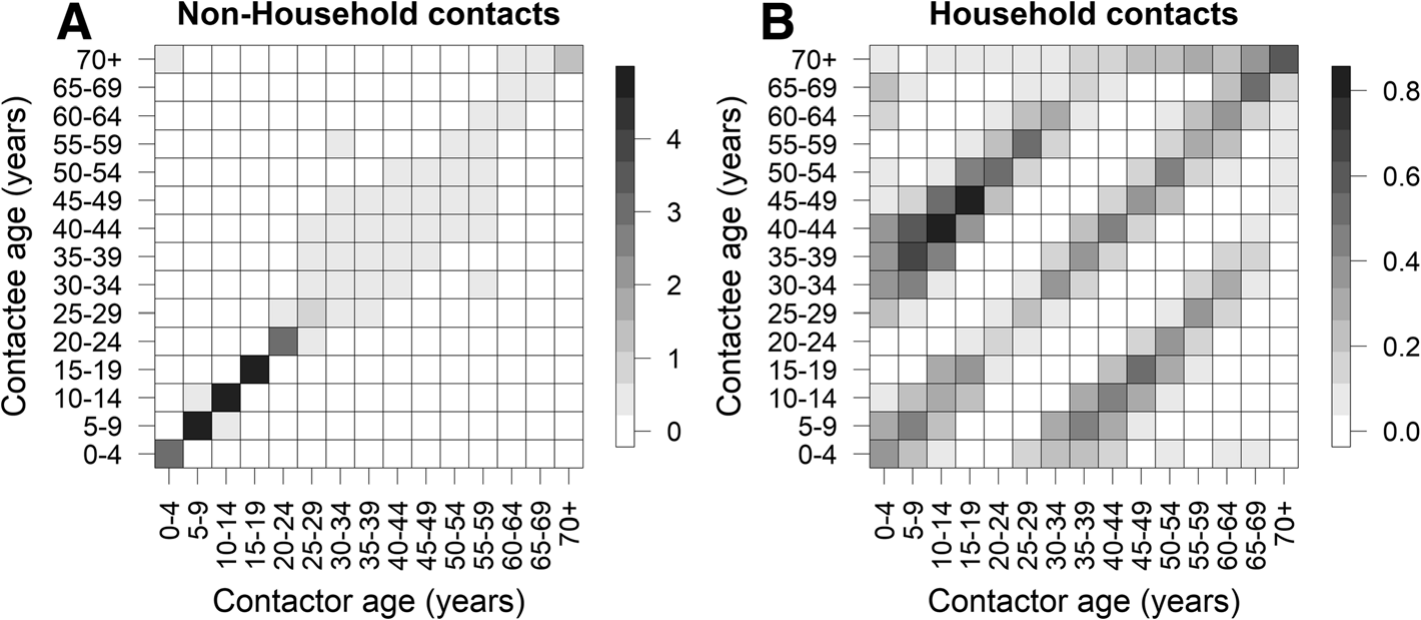
Contact matrix within the household and in the community (weekday contact). Colour bars indicate the mean number of contacts. **a** Non-household contact matrix represents the estimated mean number of contacts per day between respondents (i.e., survey participant) and persons other than their household members. **b** Household contact matrix represents the estimated mean number of contacts per day between respondents (i.e., survey participants) and their household members. Household is defined as the same unit of living space, and household members are the individuals who share that living space, regardless of blood relationship

Figure 3 shows contacts classified into those in households and the community. The non-household contacts indicate that assortative or within-age group contacts dominated the overall contacts (Fig. 3a). However, in households, contacts between parents and children dominated the overall contacts (Fig. 3b). Contacts in the community are likely to be non-physical, whereas those in households are frequently physical contacts.

**Fig. 4:**
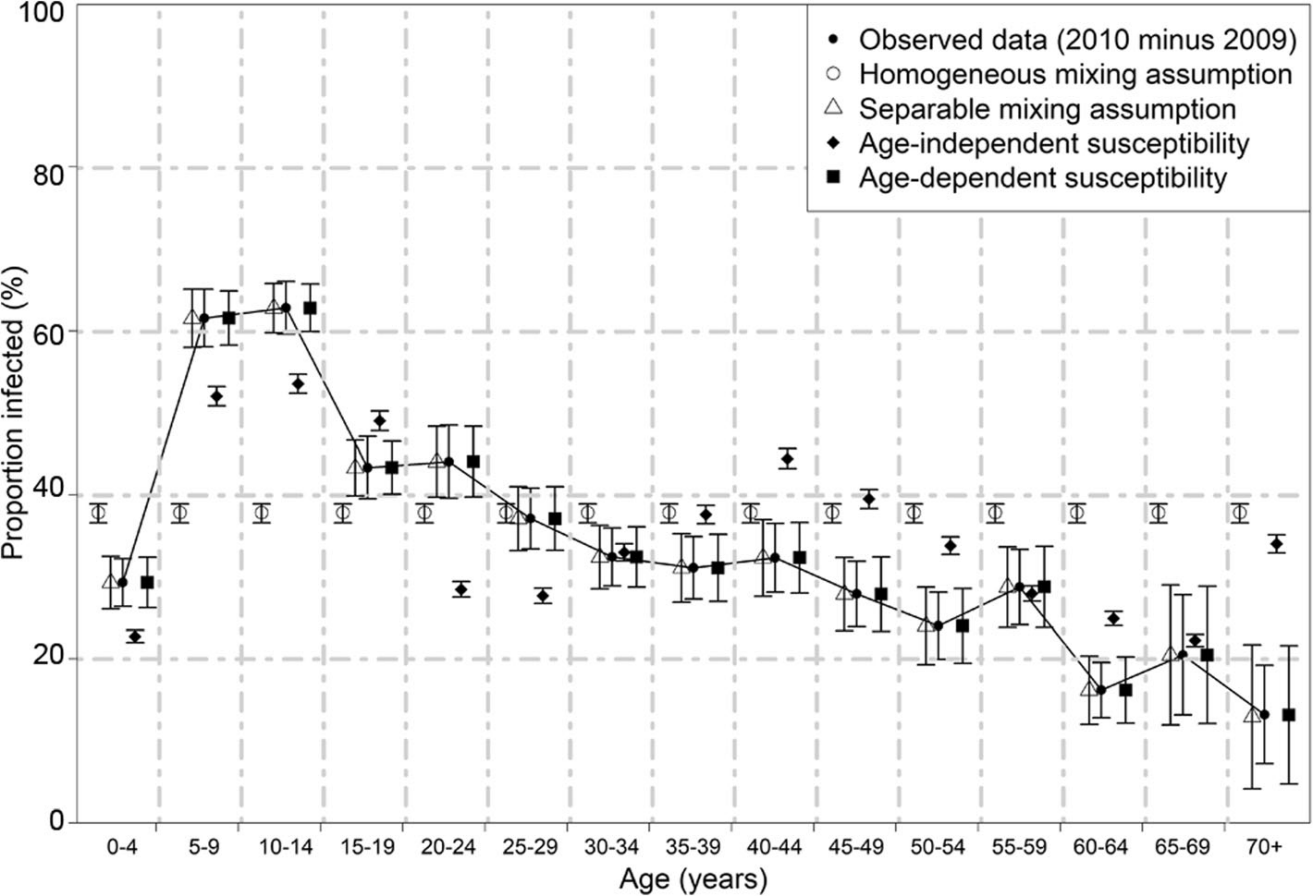
Comparison between observed and estimated age-specific proportions of infected individuals during 2009 influenza A (H1N1) pandemic. Age-specific proportions of infection, or the so-called population attack rate or final size, during the 2009 influenza A (H1N1) pandemic, illustrated by age. Estimates were obtained by imposing various assumptions of age-dependent contact patterns, including homogeneous (or random) mixing, separable mixing (i.e., contributions of contactor and contactee are separable), age-independent susceptibility (i.e., the contact matrix was used, but the entire next-generation matrix was assumed proportional to that matrix), and age-dependent susceptibility (i.e., contact matrix plus age-dependent susceptibility per contact were estimated)

Analysing the difference in the seropositive proportions between 2009 and 2010 (Additional files 1: Table S1), we estimated the next-generation matrix using the above-mentioned weekday contact matrix. Figure 4 shows a comparison of the observed fraction infected against the predicted cumulative incidence, using a variety of contact heterogeneity methods. The highest proportion infected was seen in adolescents aged 10–14 years (64.0%) and the lowest proportion was among elderly people aged 60–64 years (13.5%). The overall cumulative incidence, weighted by age-specific population, was 36.7%. Table 1 shows results from the model comparison. Without accounting for age-dependent heterogeneity, *R*_0_ was estimated at 1.25 (95% CI: 1.24, 1.26), but the AIC was as large as 819. When we accounted for age-dependent heterogeneity without using the contact matrix, the AIC was reduced, but it remained unclear whether the estimated heterogeneity sufficiently captured the age-dependent heterogeneous transmission patterns. Using the social contact matrix, but without adjusting age-dependent susceptibility, the AIC was elevated to 458. Using the matrix and adjusting for age-dependent susceptibility, a minimum AIC of 209 was obtained, and *R*_0_ was estimated to be 1.45 (95% CI: 1.42, 1.49). Owing to maximum use of the degrees of freedom, AIC for separable mixing and that using the contact matrix with adjustment of age-dependent susceptibility were the same to the second decimal place, but the latter yielded the minimum value with greater precision. On the other hand, when infectiousness was varied with age, the resulting improvement of AIC was not as large as that of age-dependent susceptibility.

**Table 1:** Comparison of model fit and estimated parameters

Contact pattern		*R*_0_^‡^ (95% confidence interval)	Number of parameters	AIC^†^
Use social contact matrix	Plus age-independent susceptibility	1.34 (1.33, 1.35)	1	457.5
	Plus age-dependent susceptibility	1.45 (1.42, 1.49)	15	208.6
Separable mixing		1.40 (1.38, 1.44)	15	208.6
Homogeneous mixing		1.25 (1.24, 1.26)	1	818.9

† Akaike information criterion, calculated as the 2NLL + 2param where NLL and param represent negative log-likelihood and the number of parameters, respectively

‡ Basic reproduction number, *R*_0_, interpreted as the average number of secondary cases generated by a single primary case in a fully susceptible population. When the age-dependent heterogeneity is taken into account, *R*_0_ is calculated as the dominant eigenvalue of the next-generation matrix.

**Fig. 5:**
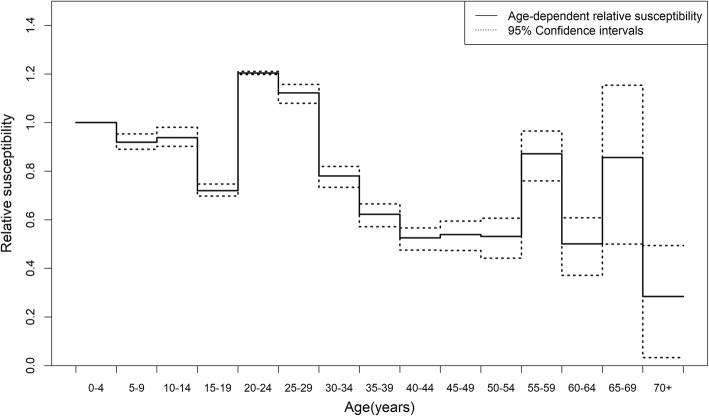
Age-dependent relative susceptibility against the 2009 influenza A (H1N1). Maximum likelihood estimates of the age-dependent relative susceptibility are shown, taking the age group 0–4 years as the reference group with the value 1.0. Dashed lines represent lower and upper 95% confidence intervals derived from the profile likelihood

Figure 5 shows the estimated age-dependent relative susceptibility, indicating a critical need to not rely on the reported contact; age-dependent susceptibility must be considered, to capture the observed age-dependent heterogeneous patterns of transmission. Larger estimates of relative susceptibility were obtained among those aged from 20 to 29 years compared with children, perhaps reflecting limited representation of the social contact among adults to capture the actual transmission of influenza. There were large variations in relative susceptibility estimates among elderly, but it is plausible that those variations were induced by sampling error.

## Discussion

In the present study, we performed a social contact survey in Japan, quantifying an age-dependent contact matrix by age group and also examining the characteristics according to location of contact. Weekday contacts were frequently seen in schools or workplaces whereas weekend contacts mostly took place in households. We identified strong age-dependent assortativity, especially among children, implying the potential effectiveness of school closure. A high rate of contact was also identified between school-age children and the age groups of their parents or guardians. The contact matrix was used to quantify heterogeneous transmission pattern by age, and we validated its usefulness by analysing seroepidemiological datasets of influenza A (H1N1) 2009. Using the social contact data, we obtained the minimum AIC, yielding an estimate of *R*_0_ for Japan at 1.45 (95% CI: 1.42, 1.49).

There have been numerous studies conducted on social contact [2, 6–13, 17–23, 30–60], including an earlier study in Japan [17] and a systematic review [30]. In the present study, we aimed to validate social contacts in Japan using infectious disease data. We specifically investigated age-dependent cumulative incidence using seroepidemiological data and estimating a next-generation matrix, which would be key to real-time interpretation of epidemiological dynamics [61–64]. We thereby demonstrated the importance of accounting for age-dependent susceptibility. It is important to consider variations by survey setting and method. In fact, depending on geographic location, a statistical study using a Bayesian hierarchical model demonstrated that the assortative contact pattern can vary by location of contact [31] (e.g., age-dependent assortativity in workplaces was the least assortative, and inter-generational contact was more frequent in Asia). Keeping in mind the possibility of such variations, the present study focused on the use of social contact data that were validated using epidemiological data of influenza, which has been missing in the literature.

The present study findings support known evidence from the POLYMOD surveys. First, in our study and also in other published studies, age-dependent contact was highly assortative, and quantifying this pattern was key to appropriately estimating age-dependent interventions, including age-dependent supplementary immunization. Second, physical and non-physical contacts were surveyed, and we observed that physical contacts were common in households whereas non-physical contacts were frequent in schools and workplaces. Physical contact may be the key to understanding transmission via contact (e.g., household transmission of Ebola virus disease), whereas non-physical contact may mirror the pattern of respiratory diseases. Different patterns between weekends and weekdays highlight the possible effectiveness of school closure on influenza transmission [32]. These are useful notions for parameterizing models to predict future incidence of infectious diseases [5].

Compared with Ibuka et al. [17], there are three major differences in our study. First, the contact matrix was validated using infectious disease data. Second, we performed age-dependent area sampling. Sampling appropriate households according to age has been identified as key for successfully capturing overall patterns of contact [37]. Third, with respect to the overall frequency of contact, the absolute number of contacts in the present study was smaller than that in Ibuka et al. [17]; the earlier study implemented a diary-based survey whereas ours relied on daily input of contacts via a website. As indicated elsewhere [37], a paper-based diary is more efficient for recording contacts than an electronic device. Improvement of the sampling scheme and data collection method remain open research subjects.

Whereas in the present study, we successfully quantified the social contact matrix, three technical issues of the social contact matrix must be noted, from the viewpoint of contact networks. First, whereas we devised the approximate matrix without individual identity, it would be useful to study how individual-based contacts affect infectious disease transmission by investigating detailed topological features as well as dynamic behaviour of the network. In addition, contact duration was not incorporated into the estimated contact matrix, which is our future aim. Second, conventional survey-based contact data collection can mostly offer only static network data and may be subject to human error, including reporting and recall biases. Moreover, such surveys may be time consuming and costly. Establishing a low-cost alternative method using novel or existing technologies is warranted [14, 16].

Four study limitations must be noted. First, the sample size of the present study was limited, with fewer than 3000 participants, smaller than that of Ibuka et al. [17]. Second, whereas we explicitly chose to use area sampling, sampling was conducted by prefecture; owing to the proportional nature, we were unable to compare differences between urban and rural locations. Third, the actual definition of contact can be very broad; however, it must be remembered that social contact surveys involve arbitrariness in defining the contact in each case. Fourth, the survey included reporting bias, e.g., respondents were mostly sampled from among homemakers registered with an internet survey company, and the extent of responses was dependent on the respondent-driven sampling of household members and respondents’ computer literacy.

We must emphasize that the present study successfully adds validated evidence of the social contact matrix to the literature, identifying common features in the published POLYMOD surveys. These datasets will be useful for parameterizing the heterogeneous transmission model of infectious diseases in Japan in the future.

## Conclusions

In the present study, we conducted a social contact survey in Japan, validating an age-dependent contact matrix according to age group and investigating the characteristics according to location of contact. Strong age-dependent assortativity was identified, especially among children, and a high rate of contact was also identified between school-age children and the age groups of their parents/guardians. Survey datasets will be useful for parameterizing the heterogeneous transmission model of various directly-transmitted infectious diseases in Japan.

## Additional files

Additional file 1: **Table S1.** Calculation of the cumulative incidence of 2009 pandemic influenza A (H1N1). (DOCX 14 kb)

Additional file 2: **Table S2.** Summary statistics of weekday contacts in Japan. (DOCX 16 kb)

Additional file 3: **Table S3.** Summary statistics of weekend contacts in Japan. (DOCX 16 kb)

Additional file 4: **Figure S1.** Proportion of physical and non-physical contacts by contact duration and location of contact (weekdays). A) Proportion of physical and non-physical contacts that took place in different locations within a day. B) Proportion of physical and non-physical contacts per day against contact duration. (TIFF 219 kb)

## Abbreviations

AIC: Akaike Information Criterion
CI: Confidence interval
