# Supplementary material for: Quantifying heterogeneous contact patterns in Japan: a social contact survey
Source: Theor Biol Med Model. 2019 Mar 20;16:6. doi: 10.1186/s12976-019-0102-8 (PMC6425701; doi:10.1186/s12976-019-0102-8)
Supplement: Supplementary file 1 — Table S1. Calculation of the cumulative incidence of 2009 pandemic influenza A (H1N1). (DOCX 14 kb) [file 12976_2019_102_MOESM1_ESM.docx]

| Before epidemic (2009) | | | | After epidemic (2010) | | | Cumulative incidence (z_i_=z_10,i_-z_9,i_) |
| --- | --- | --- | --- | --- | --- | --- | --- |
| Age group (years) | Sample  population (n_9,i_) | Number of positives (m_9,i_) | Proportion positive  (z_9,i_=m_9,i_/_19,i_) | Sample  population (n_10,i_) | Number of positives (m_10,i_) | Proportion positive (z_10,i_=m_10,i_/n_10,i_) |  |
| 0-4 | 774 | 56 | 0.07 | 770 | 269 | 0.35 | 0.28 |
| 5-9 | 527 | 31 | 0.06 | 622 | 426 | 0.68 | 0.62 |
| 10-14 | 665 | 84 | 0.13 | 726 | 556 | 0.77 | 0.64 |
| 15-19 | 508 | 173 | 0.34 | 559 | 430 | 0.77 | 0.43 |
| 20-24 | 459 | 103 | 0.22 | 410 | 271 | 0.66 | 0.44 |
| 25-29 | 539 | 85 | 0.16 | 547 | 284 | 0.52 | 0.36 |
| 30-34 | 543 | 123 | 0.23 | 564 | 303 | 0.54 | 0.31 |
| 35-39 | 492 | 70 | 0.14 | 472 | 207 | 0.44 | 0.30 |
| 40-44 | 402 | 71 | 0.18 | 399 | 194 | 0.49 | 0.31 |
| 45-49 | 412 | 80 | 0.19 | 397 | 181 | 0.46 | 0.27 |
| 50-54 | 365 | 55 | 0.15 | 340 | 126 | 0.37 | 0.22 |
| 55-59 | 342 | 25 | 0.07 | 308 | 106 | 0.34 | 0.27 |
| 60-64 | 213 | 18 | 0.08 | 337 | 74 | 0.22 | 0.14 |
| 65-69 | 146 | 12 | 0.08 | 91 | 24 | 0.26 | 0.18 |
| 70 + | 152 | 26 | 0.02 | 84 | 23 | 0.27 | 0.25 |

Table S1. Calculation of the cumulative incidence of 2009 pandemic influenza A(H1N1)
