# Supplementary material for: Quantifying heterogeneous contact patterns in Japan: a social contact survey
Source: Theor Biol Med Model. 2019 Mar 20;16:6. doi: 10.1186/s12976-019-0102-8 (PMC6425701; doi:10.1186/s12976-019-0102-8)
Supplement: Supplementary file 2 — Table S2. Summary statistics of weekday contacts in Japan. (DOCX 16 kb) [file 12976_2019_102_MOESM2_ESM.docx]

Table S2. Summary statistics of weekday contacts in Japan

| Age group (years) | Number of respondents | Average  contacts | Average non-physical contacts | Average physical contacts | Non-physical contacts | | | | Physical contacts | | | |
| --- | --- | --- | --- | --- | --- | --- | --- | --- | --- | --- | --- | --- |
|  |  |  |  |  | Home | School/ University | Work place | Other | Home | School/ University | Work place | Other |
| 0-4 | 33 | 5.48 | 0.12 | 5.36 | 0.03 | 0.00 | 0.00 | 0.09 | 3.00 | 0.73 | 0.00 | 1.63 |
| 5-9 | 68 | 8.41 | 3.40 | 5.01 | 0.47 | 1.79 | 0.00 | 1.14 | 2.07 | 1.68 | 0.01 | 1.25 |
| 10-14 | 88 | 7.86 | 4.27 | 3.59 | 0.98 | 2.44 | 0.00 | 0.85 | 1.47 | 1.65 | 0.00 | 0.47 |
| 15-19 | 84 | 7.38 | 5.67 | 1.71 | 1.76 | 3.27 | 0.10 | 0.54 | 0.61 | 0.94 | 0.01 | 0.15 |
| 20-24 | 51 | 5.16 | 4.32 | 0.84 | 1.08 | 1.90 | 0.88 | 0.46 | 0.41 | 0.24 | 0.00 | 0.19 |
| 25-29 | 79 | 4.91 | 4.04 | 0.87 | 0.78 | 0.33 | 2.41 | 0.52 | 0.47 | 0.08 | 0.16 | 0.16 |
| 30-34 | 149 | 4.56 | 3.10 | 1.46 | 0.67 | 0.05 | 1.43 | 0.95 | 0.85 | 0.01 | 0.23 | 0.37 |
| 35-39 | 193 | 4.95 | 3.45 | 1.50 | 0.69 | 0.05 | 1.86 | 0.85 | 1.20 | 0.01 | 0.12 | 0.17 |
| 40-44 | 268 | 5.24 | 3.68 | 1.56 | 0.82 | 0.10 | 1.83 | 0.93 | 1.22 | 0.01 | 0.18 | 0.15 |
| 45-49 | 296 | 4.97 | 3.86 | 1.11 | 1.15 | 0.02 | 1.90 | 0.79 | 0.79 | 0.00 | 0.16 | 0.16 |
| 50-54 | 254 | 5.02 | 4.12 | 0.90 | 1.27 | 0.04 | 1.90 | 0.91 | 0.63 | 0.00 | 0.12 | 0.15 |
| 55-59 | 186 | 5.04 | 4.10 | 0.94 | 1.10 | 0.01 | 2.03 | 0.96 | 0.47 | 0.01 | 0.18 | 0.28 |
| 60-64 | 126 | 4.52 | 3.75 | 0.77 | 1.12 | 0.00 | 1.36 | 1.27 | 0.46 | 0.00 | 0.10 | 0.21 |
| 65-69 | 94 | 3.90 | 3.21 | 0.69 | 1.14 | 0.00 | 0.63 | 1.44 | 0.31 | 0.00 | 0.05 | 0.33 |
| 70 + | 86 | 3.55 | 2.74 | 0.81 | 1.27 | 0.00 | 0.05 | 1.42 | 0.37 | 0.00 | 0.00 | 0.44 |
