# Supplementary material for: Quantifying heterogeneous contact patterns in Japan: a social contact survey
Source: Theor Biol Med Model. 2019 Mar 20;16:6. doi: 10.1186/s12976-019-0102-8 (PMC6425701; doi:10.1186/s12976-019-0102-8)
Supplement: Supplementary file 3 — Table S3. Summary statistics of weekend contacts in Japan. (DOCX 16 kb) [file 12976_2019_102_MOESM3_ESM.docx]

Table S3. Summary statistics of weekend contacts in Japan

| Age group (years) | Number of respondents | Average  contacts | Average non-physical contacts | Average physical contacts | Non-physical contacts | | | | Physical contacts | | | |
| --- | --- | --- | --- | --- | --- | --- | --- | --- | --- | --- | --- | --- |
|  |  |  |  |  | Home | School/ University | Work place | Other | Home | School/ University | Work place | Other |
| 0-4 | 33 | 4.15 | 0.27 | 3.88 | 0.09 | 0.00 | 0.00 | 0.18 | 2.97 | 0.00 | 0.00 | 0.91 |
| 5-9 | 68 | 5.57 | 1.32 | 4.25 | 0.32 | 0.13 | 0.00 | 0.87 | 2.51 | 0.31 | 0.00 | 1.43 |
| 10-14 | 89 | 5.42 | 2.18 | 3.24 | 1.12 | 0.43 | 0.00 | 0.63 | 1.97 | 0.45 | 0.00 | 0.82 |
| 15-19 | 83 | 5.35 | 3.98 | 1.37 | 1.86 | 1.02 | 0.10 | 1.00 | 0.78 | 0.12 | 0.02 | 0.45 |
| 20-24 | 51 | 4.16 | 3.28 | 0.88 | 1.16 | 0.75 | 0.35 | 1.02 | 0.53 | 0.00 | 0.00 | 0.35 |
| 25-29 | 80 | 3.73 | 2.55 | 1.18 | 0.91 | 0.13 | 0.44 | 1.07 | 0.61 | 0.04 | 0.04 | 0.49 |
| 30-34 | 153 | 3.67 | 2.12 | 1.55 | 0.68 | 0.08 | 0.51 | 0.85 | 1.08 | 0.01 | 0.07 | 0.39 |
| 35-39 | 195 | 4.26 | 2.48 | 1.78 | 0.65 | 0.05 | 0.54 | 1.24 | 1.37 | 0.01 | 0.09 | 0.31 |
| 40-44 | 268 | 4.24 | 2.44 | 1.80 | 0.85 | 0.08 | 0.28 | 1.23 | 1.45 | 0.00 | 0.04 | 0.31 |
| 45-49 | 294 | 4.03 | 2.81 | 1.22 | 1.13 | 0.04 | 0.49 | 1.15 | 0.95 | 0.00 | 0.04 | 0.23 |
| 50-54 | 267 | 4.29 | 3.26 | 1.03 | 1.34 | 0.03 | 0.62 | 1.27 | 0.73 | 0.00 | 0.05 | 0.25 |
| 55-59 | 191 | 3.94 | 2.93 | 1.01 | 1.24 | 0.00 | 0.43 | 1.26 | 0.54 | 0.00 | 0.12 | 0.35 |
| 60-64 | 135 | 4.11 | 3.28 | 0.83 | 1.46 | 0.00 | 0.36 | 1.46 | 0.53 | 0.00 | 0.07 | 0.23 |
| 65-69 | 97 | 4.23 | 3.25 | 0.98 | 1.20 | 0.00 | 0.38 | 1.67 | 0.51 | 0.00 | 0.05 | 0.42 |
| 70 + | 91 | 3.74 | 2.91 | 0.83 | 1.36 | 0.00 | 0.02 | 1.53 | 0.46 | 0.00 | 0.00 | 0.37 |
