# Supplementary figures and images for: Quantifying heterogeneous contact patterns in Japan: a social contact survey
Source: Theor Biol Med Model. 2019 Mar 20;16:6. doi: 10.1186/s12976-019-0102-8 (PMC6425701; doi:10.1186/s12976-019-0102-8)

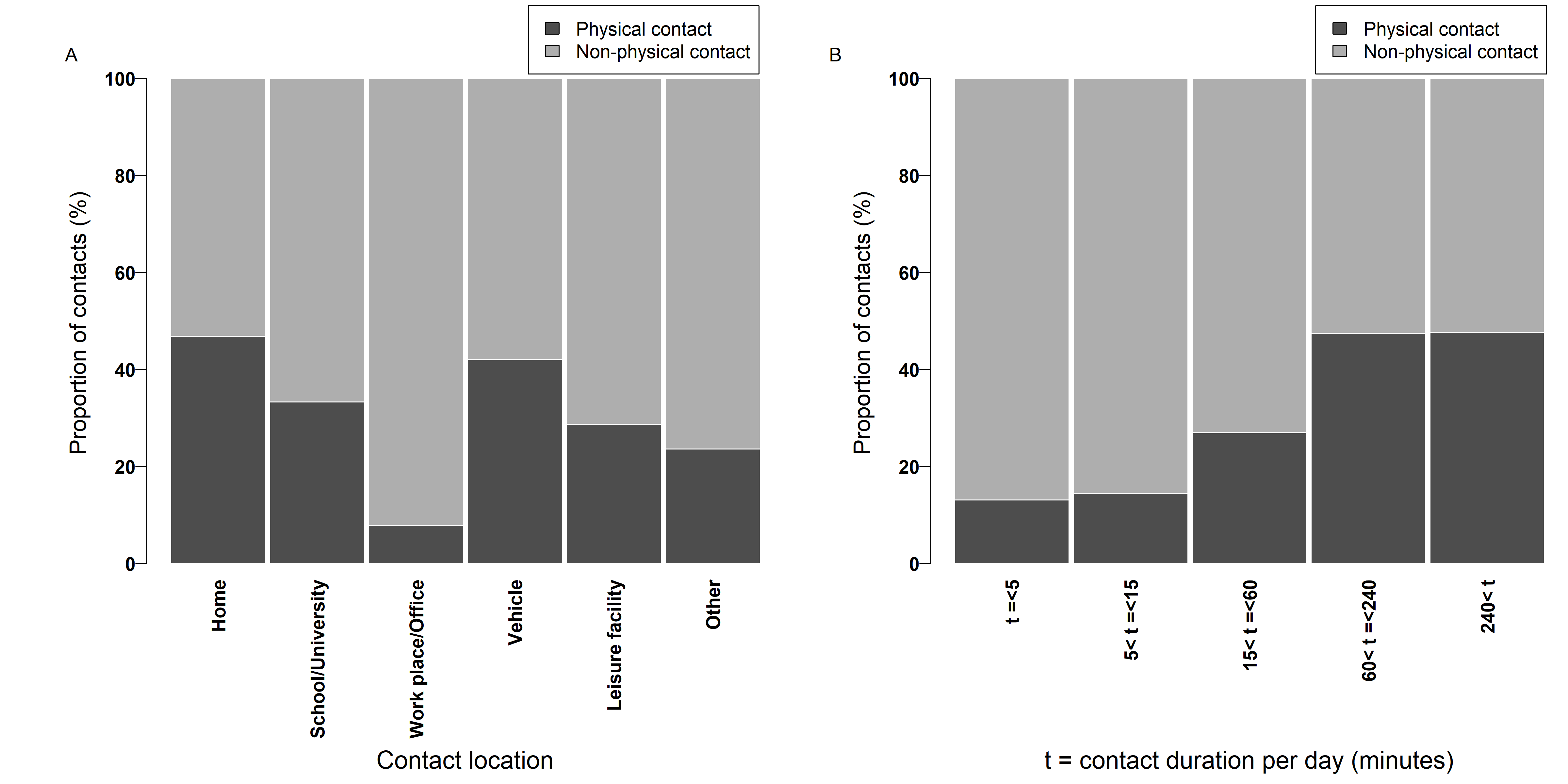

Supplement: Supplementary file 4 — Figure S1. Proportion of physical and non-physical contacts by contact duration and location of contact (weekdays). A) Proportion of physical and non-physical contacts that took place in different locations within a day. B) Proportion of physical and non-physical contacts per day against contact duration. (TIFF 219 kb) [file 12976_2019_102_MOESM4_ESM.tiff]
